# The Viable Microbiome of Human Milk Differs from the Metataxonomic Profile

**DOI:** 10.3390/nu13124445

**Published:** 2021-12-13

**Authors:** Lisa F. Stinson, Michelle L. Trevenen, Donna T. Geddes

**Affiliations:** 1 School of Molecular Sciences, The University of Western Australia, Crawley 6009, Australia; donna.geddes@uwa.edu.au; 2 Centre for Applied Statistics, The University of Western Australia, Crawley 6009, Australia; michelle.trevenen@uwa.edu.au

**Keywords:** human milk, microbiome, viability, propidium monoazide (PMA), breastfeeding

## Abstract

Bacteria in human milk contribute to the establishment of the infant gut microbiome. As such, numerous studies have characterized the human milk microbiome using DNA sequencing technologies, particularly 16S rRNA gene sequencing. However, such methods are not able to differentiate between DNA from viable and non-viable bacteria. The extent to which bacterial DNA detected in human milk represents living, biologically active cells is therefore unclear. Here, we characterized both the viable bacterial content and the total bacterial DNA content (derived from viable and non-viable cells) of fresh human milk (*n* = 10). In order to differentiate the living from the dead, a combination of propidium monoazide (PMA) and full-length 16S rRNA gene sequencing was used. Our results demonstrate that the majority of OTUs recovered from fresh human milk samples (67.3%) reflected DNA from non-viable organisms. PMA-treated samples differed significantly in their bacterial composition compared to untreated samples (PERMANOVA *p* < 0.0001). Additionally, an OTU mapping to *Cutibacterium acnes* had a significantly higher relative abundance in PMA-treated (viable) samples. These results demonstrate that the total bacterial DNA content of human milk is not representative of the viable human milk microbiome. Our findings raise questions about the validity of conclusions drawn from previous studies in which viability testing was not used, and have broad implications for the design of future work in this field.

## 1. Introduction

The human milk microbiome has been extensively characterized using both cultivation-based and DNA-based techniques [1]. However, neither of these methods are able to provide information on both the viable and non-viable bacterial content of human milk. Bacterial culture relies on bacterial growth, and is therefore unable to detect non-reproducing or non-viable bacterial cells. Bacterial culture is also limited by the existence of viable but non-culturable (VBNC) bacteria [2]. DNA sequencing techniques detect both viable and non-viable bacteria, but are unable to differentiate between DNA from living and dead cells. Therefore, the extent to which bacterial DNA detected in milk “microbiome” studies reflects a true, living microbiome is unclear. While DNA-based metataxonomic studies of the human milk microbiome report a large diversity of bacterial taxa [3,4,5], numerous studies have failed to culture bacteria from individual milk samples, or have successfully cultured only a small number of taxa [6,7,8]. This discrepancy may suggest that many of the bacteria detected in human milk using DNA-based techniques are non-viable.

Given that human milk contains a very low biomass of bacteria [9,10,11], it may be that this substance suppresses bacterial growth. Indeed, human milk contains a range of anti-microbial compounds, including lactoferrin, lysozyme, immunoglobulins, and immune cells [12,13,14]. These factors may act to prevent overgrowth of bacteria in the lactating mammary gland. Further, the low titers of bacteria in human milk may prevent the immature infant immune system from becoming overwhelmed. Ultimately, a breastfed infant likely consumes both viable and non-viable bacteria, both of which may have distinct biological functions. It is therefore important to characterize the viability of bacteria detected using common 16S rRNA gene sequencing techniques to better understand the bacterial composition of human milk.

Here, we aimed to characterize both the viable bacterial content of human milk, and the total bacterial DNA content (derived from viable and non-viable cells). In order to delineate DNA from viable and non-viable organisms, a combination of propidium monoazide (PMA; a DNA chelating agent that is excluded by viable cell membranes [15]) and full-length 16S rRNA gene sequencing was used.

## 2. Materials and Methods

### 2.1. Sample Collection

Milk samples were collected from lactating women (*n* = 10; 1–12 months post-partum) who attended study sessions at The University of Western Australia. This study was approved by the University of Western Australia’s Human Research Ethics Committee (RA/4/1/2369) and all participants provided informed consent. All participants were healthy and had not taken antibiotics within the past month. Participants were asked not to express or breastfeed from the breast they had elected to donate milk from for at least 2 h before providing the sample. Participants expressed a 50 mL sample using a Symphony electric breast pump and a sterile pump kit (Medela AG, Baar, Switzerland). Samples were immediately transported to the lab for processing (<30 min, room temperature). Two 1 mL aliquots from each sample were analyzed: one with a PMA pre-treatment to assess the viable microbiome, and one without PMA to assess the total bacterial DNA profile. Certified DNA and DNase-free tubes were used for all processing steps (Sarstedt).

### 2.2. PMA Treatment

Human milk samples were centrifuged at 10,000× *g* for 10 min at 4 °C, and the fat and supernatant were discarded. Although we have previously reported that bacteria are present in the fat content of human milk [9], it was necessary to remove the fat to allow light penetration of the sample. For the PMA-treated aliquots, samples were treated with PMA (PMAxx™, Biotium, Fremont, CA, USA) immediately prior to DNA extraction following the manufacturer’s protocol. Briefly, samples were topped up to 500 µL with nuclease-free water (Integrated DNA Technologies, Inc., Coralville, IA, USA). 1.25 µL of 20 mM PMAxx™ was added to each sample to a final concentration of 50 µM. Samples were vortexed for 20 s then incubated in the dark at 37 °C for 15 min with vortexing every 5 min. Samples were then exposed to light using a PMA-Lite LED Photolysis Device (Biotium, Fremont, CA, USA) for 15 min, with vortexing every 5 min to ensure all elements of the sample were exposed to light. Cells were pelleted by centrifuging at 5000× *g* for 10 min and the supernatant discarded. DNA was then immediately extracted from the cell pellet. For the non-PMA treated aliquots, samples were kept at room temperature for the duration of the PMA-treatment of the paired aliquots. DNA was extracted from the cell pellets alongside the PMA-treated aliquots.

### 2.3. DNA Extraction

DNA was extracted using the QIAGEN MagAttract Microbial DNA kit on the King Fisher Duo platform according to the manufacturer’s instructions. A negative extraction control consisting of reagents only was included in each batch (*n* = 4). Eluates were stored at −20 °C until analysis. Total DNA yield was assessed using a Qubit high sensitivity dsDNA kit on a Qubit 2.0 Fluorometer (ThermoFisher, Waltham, MA, USA). The limit of detection was 10 pg/µL.

### 2.4. PacBio Sequencing

The full-length 16S rRNA gene was amplified using the primers 27F (5′-AGRGTTYGATYMTGGCTCAG-3′) and 1492R (5′-RGYTACCTTGTTACGACTT-3′), as previously described [16]. Primers were tagged with the universal sequences UNITAG-R (tggatcacttgtgcaagcatcacatcgtag) and UNITAG-F (gcagtcgaacatgtagctgactcaggtcac) (ligated to the 5′end of the primers). A set of eight barcoded UNITAG-F and 15 barcoded UNITAG-R primers were designed to generate PacBio sequencing-ready amplicons, using an asymmetric barcoding strategy. PCR was carried out in two rounds. In the first, template was amplified in 30 µL reactions, made up of 6 µL template, 0.3 µM each for forward and reverse primers, 0.75 µL each for ArctriZymes dsDNase and DTT, and 6.6 µL of water. Amplification was carried out in a Veriti Thermal Cycler. The program consisted of an initial heating stage of 94 °C for 3 min followed by 35 cycles of 94 °C for 30 s, 55 °C for 30 s, and 72 °C for 2 min. A no template control was included to assess potential contamination introduced by the PCR reagents. The PCR products were verified by size on a QIAXcel automated electrophoresis system using a DNA high resolution gel cartridge. Amplicons were purified using Macherey-Nagel NucleoMag magnetic beads.

Barcoding of primary amplicons was performed in a secondary amplification procedure. First, 25 µL reactions were made up of 12.5 µL AccuStart II PCR ToughMix, 20 µM each of the forward and reverse primers, 5 µL of template and 5.5 µL of water. The same cycling conditions were used as in the first amplification, with eight cycles performed. Barcoded samples were pooled in an equimolar concentration and the pool was made up to 50 µL with TE buffer. Finally, the pool was gel purified using the QIAquick Gel Extraction Kit.

The purified, barcoded DNA pool sequenced at the Australian Genome Research Facility (University of Queensland, Brisbane, QLD, Australia) using the PacBio Sequel II system.

### 2.5. Sequence Processing

PacBio raw reads were demultiplexed to obtain circular consensus sequence (CCS) reads for each sample. CCS reads were filtered to retain only those with a minimum of three full passes and 99.9% sequence accuracy. Sequence data was processed using mothur version 1.44.1 [17]. Sequences were length filtered (1336–1743 bp) and filtered for sequences containing homopolymers of >9 bases. Alignment was performed using the SILVA reference alignment database (v138). Chimeric sequences and those mapping to non-bacterial taxa were removed. Sequences were clustered into OTUs by first calculating the pairwise distances between sequences using the dist.seqs command, followed by clustering with a similarity cutoff of 0.03 using the cluster command. Subsampling was performed to 1592 reads based on the size of the smallest library (not including negative controls). Taxa identified in negative controls are reported in Supplementary Table S1.

### 2.6. Data Availability

FASTQ sequences have been deposited to NCBI SRA (PRJNA758749).

### 2.7. Statistical Analysis

#### 2.7.1. DNA Quantification

The distribution of DNA quantity was visually inspected and as the distribution was right-skewed analysis was performed after a log-transformation. Medians and inter-quartile ranges (IQR) are reported. A linear mixed model was performed with an outcome of (log-transformed) DNA quantity, a fixed factor of PMA treatment, and a random effect of participant ID to take into account the paired nature of the data. Pairwise comparisons were back-transformed to ratios, with associated 95% confidence intervals (CIs) and *p*-values, such that interpretation may be on the original DNA quantity scale. There was one outlier, so analyses were performed on both the full dataset as well as with that outlier removed. Significance was considered at the 5% level for this and all following analyses.

#### 2.7.2. Alpha Diversity

Alpha diversity was assessed using Shannon diversity and richness (number of OTUs). The distribution of these were examined through visual inspection. The Shannon diversity appeared normally distributed, whilst richness was right skewed. As such, means and standard deviations (SDs) are reported for Shannon diversity, whilst medians and IQRs are reported for richness. Linear mixed models were performed with outcomes of Shannon diversity and log-transformed richness. A fixed effect of PMA treatment and a random effect of participant ID, to take into account the paired nature of the data, were included in the models. Estimated mean differences are provided for the Shannon diversity analysis and back-transformed ratios for the richness analysis, in addition to the associated 95% CIs and *p*-values.

#### 2.7.3. Beta Diversity

Beta diversity was assessed by performing a PERMANOVA using Bray-Curtis distances. A fixed effect of PMA treatment and a random effect of participant ID, to take into account the paired nature of the data, were included in the model. *p*-values are provided. In order to visualize the Bray-Curtis distances, a principal co-ordinates analysis (PCoA) ordination was performed for two dimensions and plotted.

#### 2.7.4. Comparison of OTU Relative Abundance

For the relative abundance analysis, results from OTUs which made up ≥1% of the total relative abundance in the samples, in conjunction with a prevalence threshold of more than 10%, are reported. Analysis was performed at the OTU level, and taxonomic assignments for each OTU were established using BLAST [18]. Relative abundances were analyzed using generalized additive models for location, scale, and shape. This is a framework for fitting regression type models that allows the response variable, in this case relative abundance, to follow any distribution. We allowed the relative abundances to follow a zero-inflated beta distribution, as this has been shown to be appropriate for this type of data [19]. PMA treatment was considered as a fixed effect and participant ID, to take into account the paired nature of the data, was included as a random effect.

## 3. Results

### 3.1. PMA-Treated Milk Contains a Significantly Lower Concentration of DNA

**Figure 1:**
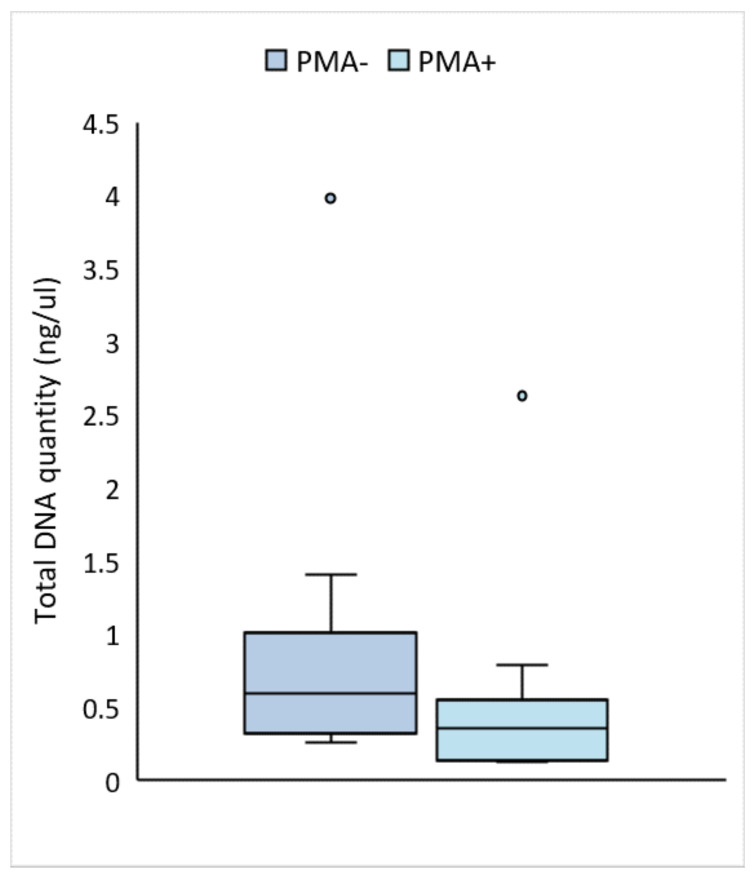
DNA concentration (ng/µL) was significantly lower in PMA-treated than untreated human milk samples (linear mixed model of log-transformed data; *p* < 0.0001). The dark blue bar represents total DNA from viable and non-viable cells (non-PMA treated samples). The light blue bar represents DNA from viable cells only (PMA-treated samples). Boxes are interquartile range, whiskers are range, and inner lines are medians.

PMA-treated samples yielded significantly less total DNA than untreated samples, suggesting that a significant portion of the DNA in human milk originates from non-viable cells (median PMA-treated: 0.36 ng/µL (IQR 0.34 ng/µL); median untreated: 0.60 ng/µL (IQR 0.55 ng/µL); estimated ratio of untreated to treated: 2.02, 95% CI: 1.67–2.45, *p* < 0.0001) (Figure 1). This relationship holds even with the removal of the outlier (estimated ratio of untreated to treated: 2.09, 95% CI 1.71–2.55, *p* < 0.0001).

### 3.2. The Viable Microbiome Differs from the Total Bacterial DNA Profile in Human Milk

**Figure 2:**
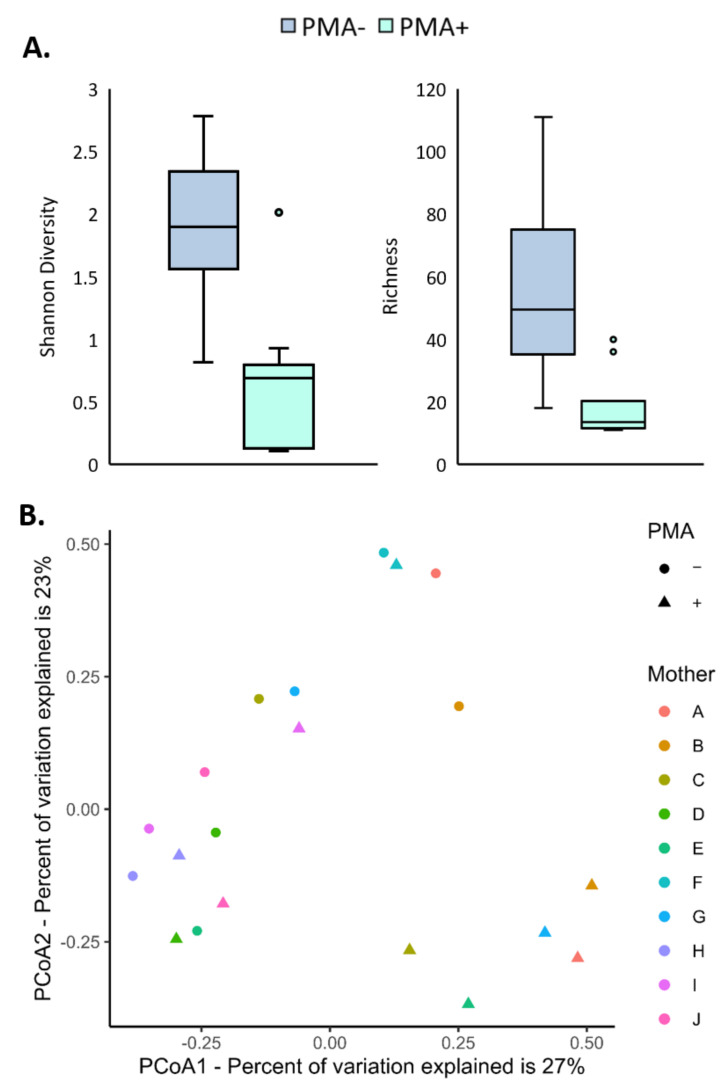
(**A**) Shannon diversity and richness of PMA-treated human milk samples was significantly lower than untreated samples (Shannon diversity: linear mixed model, *p* = 0.0188; richness: linear mixed model of log-transformed data, *p* = 0.0001). Boxes are interquartile range, whiskers are range, and inner lines are medians. Light blue boxes are PMA-treated samples, while dark blue boxes are untreated samples. (**B**) PCoA plot of Bray-Curtis distances of PMA-treated and untreated human milk samples. Different colors denote different individuals, while shape denotes PMA treatment status (triangle is treated and circle is untreated).

Shannon diversity was significantly reduced in PMA-treated samples (mean in untreated samples: 1.89 (SD 0.53), mean in treated samples: 1.36 (SD 0.61), estimated mean difference: 0.53, 95% CI: 0.11–0.95, *p* = 0.0188) (Figure 2A). Richness was also significantly reduced in PMA-treated samples (median number of OTUs in untreated samples: 49.5 (IQR 33), median number of OTUs in PMA-treated samples: 13.5 (IQR 3); estimated ratio of untreated to treated samples: 3.06, 95% CI: 2.08–4.48, *p* = 0.0001) (Figure 2A). This demonstrates that a significant number of OTUs identified in these milk samples were non-viable. At the community level, the bacterial composition of PMA-treated samples differed significantly from untreated samples (PERMANOVA *p* < 0.0001) (Figure 2B).

**Figure 3:**
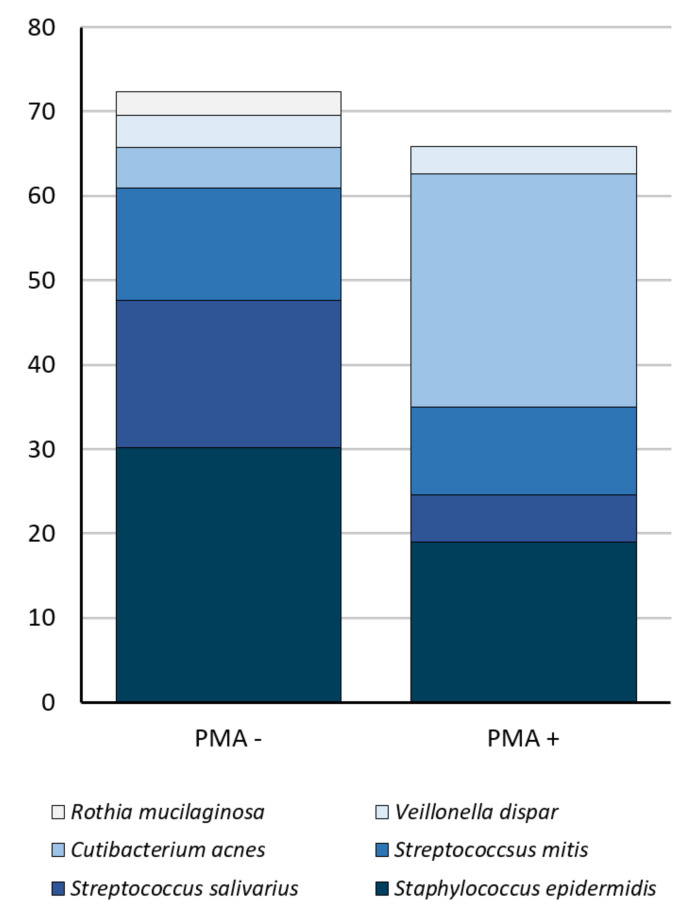
Bacterial DNA profiles differed in PMA-treated (PMA+) and untreated (PMA-) samples. The relative abundance (%) of the six OTUs occurred at a relative abundance of ≥1% and prevalence of ≥10% are shown here.

**Figure 4:**
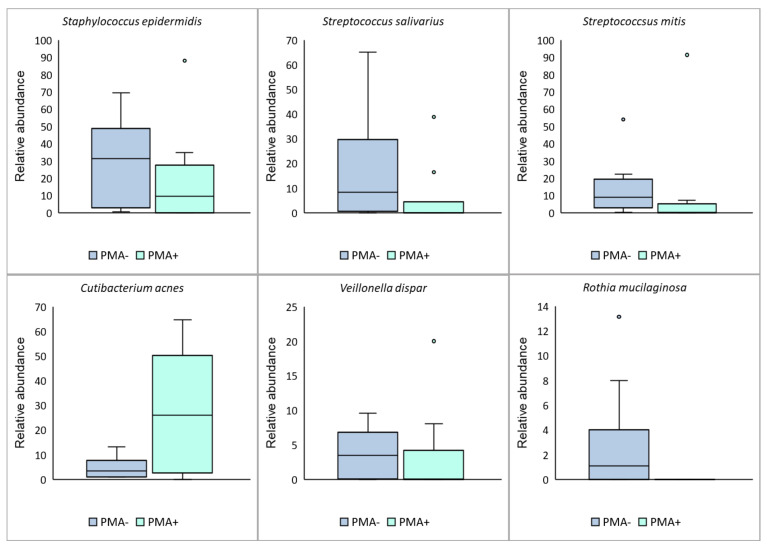
PMA treatment resulted in changes to the relative abundance of the top six OTUs (≥1% relative abundance, ≥10% prevalence) in human milk samples. The dark blue bar represents total DNA from viable and non-viable cells (non-PMA treated samples). The light blue bar represents DNA from viable cells only (PMA-treated samples). Boxes are interquartile range, whiskers are range, and inner lines are medians. *Cutibacterium acnes* relative abundance was significantly higher in PMA-treated compared to untreated samples (generalized additive model for location, scale and shape fit with a zero-inflated beta distribution; *p* = 0.0017).

Compositional differences were also seen at the OTU level. Overall, six OTUs made up ≥1% total relative abundance and >10% prevalence within these samples (Figure 3 and Figure 4). Of these, one OTU, mapping to *Cutibacterium acnes*, had a significantly higher relative abundance in PMA-treated samples than in untreated samples (*p* = 0.0017). While this OTU made up only a small fraction of the total bacterial DNA profile (4.8%), it was the dominant OTU in PMA-treated samples (27.6% relative abundance) (Figure 4). The reduction in relative abundance of these OTUs in PMA-treated samples may suggest that they consist of a large proportion of non-viable organisms. Similarly, one OTU, which mapped to *Rothia mucilaginosa*, was present in untreated samples, but absent from PMA-treated samples, suggesting that none of the DNA from this organism had originated from viable cells (Figure 3 and Figure 4).

## 4. Discussion

This is the first study of the human milk microbiome to use PMA to exclude DNA from non-viable bacterial cells. An important finding of this study is that the majority of OTUs detected in fresh human milk by 16S rRNA gene sequencing are non-viable. The richness of viable bacteria in human milk is therefore far lower than what has previously been reported using DNA sequencing methods. Numerous culture-based studies of the human milk microbiome have failed to detect bacteria in individual samples, or have detected only a low number of organisms [6,7,8]. Conversely, metataxonomic studies of the human milk microbiome repeatedly report the presence of a diverse range of organisms [3,4,5]. The data presented here may explain this discrepancy. This emphasizes the need for viability testing to be coupled with standard bacterial sequencing techniques in order to interrogate the true, living human milk microbiome. These findings are not limited to studies of human milk. Misrepresentation of the viable microbiome using DNA-based techniques is likely to impact other sample types. Indeed this “viability bias” has been demonstrated using PMA for infant stool samples [20,21], cleanroom samples [22,23], and even samples taken from the international space station [24].

PMA-treated samples (DNA from intact, viable cells) also differed from untreated samples in terms of their composition (beta diversity and relative abundance of individual OTUs). In non-PMA treated samples, we observed a dominance of *Staphylococcus* spp. and *Streptococcus* spp., with *Staphylococcus epidermidis* predominating (30.2% average relative abundance); as has been repeatedly observed in 16S rRNA gene sequencing studies of human milk [3,4,5,9,25]. However, within PMA-treated samples, *Cutibacterium acnes* was the dominant species (27.6% average relative abundance, compared to 4.8% in untreated samples). This species is often overlooked in descriptions of the “core” human milk microbiome; however, these data suggest that it may be a major viable taxon in this sample type. Interestingly, typical oral taxa, such as *Streptococcus salivarius*, *Streptococcus mitis*, and *R. mucilaginosa* were less abundant in PMA-treated compared to untreated samples. This may suggest that these taxa, which likely originate from the infant oral cavity [1,26], may not survive transfer to the breast. Collectively, these results emphasize the fact the DNA sequencing alone does not accurately represent the viable human milk microbiome. This has implications for interpretation of previous studies, as well as design of future studies, by emphasizing the need for viability testing. However, given that this is the first study to utilize viability testing, further work examining the contribution of viable and non-viable bacteria to the total milk bacterial DNA profile is required to validate the results. It is important to note that penetration of dead cell membranes by PMA may not be complete, and that partially compromised cell membranes may not permit passage of PMA [27]. The abundance of viable bacteria reported here should therefore be interpreted as maximal values, as the level of dead cells may be underestimated with PMA.

On average, approximately half (49.2%, SD 11.4%) of the total DNA yielded from fresh human milk samples derived from non-viable cells. It should be noted that for biosecurity reasons, these samples were transported at room temperature from the location in which they were expressed to the laboratory, which typically took approximately 10 min. In this time cells within the samples may have divided or become non-viable, thus impacting our results. Nevertheless, our findings on cell viability in human milk are in line with previous reports in human fecal samples, in which only 49% of bacterial cells were intact, while 19% were injured or damaged, and 32% were dead [28]. The measure of total DNA used in the current study includes both human and non-human DNA, which we have previously reported to be at a ratio of approximately 1:1 in human milk [9]. Of the human cell populations in milk, mammary epithelial cells (milk secreting cells) and myoepithelial cells (which line the ducts and alveoli) make up 60–90% [29]. Their presence in milk likely occurs when they are sloughed off from the inner layer of the mammary architecture. Their populations in milk may therefore be largely non-viable. Additionally, human milk may be a hostile environment for bacteria due to the presence of a diverse range of anti-microbial compounds and immune cells [13,14,29]. Rather than a permanent resident microbiome, it may be that exogenous bacteria migrate into the mammary gland from the maternal gut, breast skin, and infant oral cavity, where they struggle to survive, until they are transported out of the mammary gland via a milk ejection [1]. Indeed, this theory may explain why human milk contains such a low biomass of bacteria. Collectively, it is therefore unsurprising that such a large portion of the total DNA quantity in human milk originates from non-viable cells.

While this small proof of concept study has demonstrated that the metataxonomic profile of human milk is not representative of the viable microbiome, it is limited by a small sample size (*n* = 10). Given the variability between the profiles of individual samples, a larger sample size may have resulted in more significant differences in the relative abundance of individual OTUs. The extent to which the bacteria detected in human milk are viable may vary between individuals. Further, the participants in this study ranged in postpartum age (1–12 months). Given evidence that the human milk microbiome varies over this time period [30,31,32], bacterial viability may also vary. Other variables such as maternal diet, maternal health, and infant health may affect the viability of the human milk microbiota. This proof of concept study should therefore be followed by larger studies to assess these variables.

The results of this study have wide ranging implications. Importantly, these results emphasize the fact the DNA sequencing alone does not accurately represent the viable human milk microbiome. This has implications for interpretation of previous studies, as well as design of future studies, by emphasizing the need for viability testing. This work also has implications in the way that we view the role of human milk bacteria in infant health. Our results suggest that infants are exposed to a range of living and dead bacteria via human milk. While there is evidence that some of the viable taxa in milk go on to colonize the infant gut microbiome [33,34,35], the non-viable taxa may also serve a biological function in the infant gut. These bacteria, delivered along with maternal immunoglobulins, may present the developing infant immune system with an opportunity for risk-free education on the recognition of bacteria. In fact, approximately 40% of bacteria in human milk have been reported to be IgA coated [36], suggesting a biological function for such bacteria. Further work is needed to assess the role of both viable and non-viable human milk bacteria in infant health, as these likely have distinct biological functions.

## Data Availability

FASTQ sequences have been deposited to NCBI SRA (PRJNA758749).

## Supplementary Materials

The following is available online at https://www.mdpi.com/article/10.3390/nu13124445/s1, Table S1: Taxa detected in negative extraction controls (NEC) and no template PCR controls (NTC).
