# Supplementary material for: The Viable Microbiome of Human Milk Differs from the Metataxonomic Profile
Source: Nutrients. 2021 Dec 13;13(12):4445. doi: 10.3390/nu13124445 (PMC8708405; doi:10.3390/nu13124445)
Supplement: Supplementary file 1 [file nutrients-13-04445-s001.zip › nutrients-1494104-SI.pdf]

Supplementary Table S1. Taxa detected in negative extraction controls (NEC) and no template PCR controls (NTC).

Genera	NEC1	NEC2	NEC3	NEC4	NTC
Rothia	0	0	0	0	1
Cutibacterium	0	263	1	2	0
Hydrogenobacter	2	0	0	0	0
Prevotella	0	36	0	0	0
Unclassified Bacilli	0	0	1	0	0
Lactobacillus	0	0	0	0	12
Streptococcus	0	2	1	1	30
Staphylococcus	2	5	4	4	3
Finegoldia	0	1	1	0	0
Peptoniphilus	0	0	1	0	0
Cupriavidus	0	2	0	0	0
Ralstonia	12921	11471	18613	25574	53
Unclassified Burkholderiales	5	15	6	3	0
Tepidiphilus	0	0	0	290	0
Unclassified Nitrosomonadaceae	0	26	0	0	0
